# Common Variable Immunodeficiency and Hodgkin Lymphoma in a 50-Year-Old Male

**DOI:** 10.7759/cureus.58989

**Published:** 2024-04-25

**Authors:** Eshani Kishore, Frederick Gyabaah, Abhizith Deoker

**Affiliations:** 1 Internal Medicine, Texas Tech University Health Sciences Center El Paso, El Paso, USA

**Keywords:** nodular subtype hodgkin's lymphoma, lymphoma, hodgkin, common variable immunodeficiency (cvid), cvid

## Abstract

Common variable immunodeficiency (CVID) is a primary immunodeficiency with the involvement of B cells, T cells, and antigen-presenting cells. Patients with CVID are more susceptible to malignancies and bacterial infections in the gastrointestinal and respiratory tracts. We discuss a case of a 50-year-old male who presented to the emergency department with a history of intermittent abdominal pain, diarrhea, night sweats, fever, nausea, and weight loss of 40 pounds over six months. A CT of the abdomen revealed splenomegaly with several infiltrated solid nodules as well as enlarged mediastinal, hilar, periesophageal, cervical, and left supraclavicular lymph nodes, findings suggestive of lymphoma. The diagnosis of nodular lymphocyte-predominant Hodgkin lymphoma was confirmed by immunohistology, which revealed that CD20 and CD3 were both positive in small lymphocytes. Immunoglobulin (Ig) levels were low for IgG and IgM, findings highly suggestive of CVID. We want to shed light on the importance of performing the clinical workup for CVID when Hodgkin lymphoma and recurrent infections are present, as the immunodeficiency remains underdiagnosed and underreported.

## Introduction

Common variable immunodeficiency (CVID) is the most common primary immunodeficiency with a typical age of onset in early adulthood. Patients have hypogammaglobulinemia, particularly low amounts of IgG, that increases susceptibility to recurrent bacterial infections, autoimmunity, lymphoproliferation, and malignancy [1].

CVID may be due to defects in B cell development, maturation, and survival. In particular, CVID affects the transmembrane activator, calcium modulator, and cyclophilin ligand interactor (TACI) involved in the activation of the B-cell receptor and Toll-like receptor 7 and 9 molecules [2]. CVID patients can also have defects in costimulatory molecules such as CD19, CD20, CD21, and CD81, and IL-21 that promote B cell function [3]. Patients with CVID may have deficits in T-cell production as well, particularly fewer total, memory, and naïve CD4+ cells [4]. Patients with lower quantities of these cells also have more severe autoimmunity and more advanced lymphoproliferation. Some patients may have lower numbers of Th_17_ cells, which increases susceptibility to bacterial and fungal infections [5].

Due to their impaired immune response, CVID patients most commonly develop infections of the respiratory tract such as bronchitis, sinusitis, and pneumonia [6]. Encapsulated organisms (*Streptococcus pneumoniae *and *Haemophilus influenzae*) or viral pathogens such as rhinovirus, coronavirus, and influenza virus are most commonly implicated [7,8]. These patients are also diagnosed with autoimmune conditions at higher rates [9]. Moreover, those living with CVID have an increased risk of malignancy, particularly lymphoma and gastric cancer. The age of first symptom onset may play a role, as the incidence of malignancy is greater when the condition is diagnosed at a later age [10]. Any CVID patient with preexisting polyclonal lymphadenopathy also has an increased risk of developing lymphoid malignancy [11].

Analysis of primary immunodeficiency epidemiology reveals that CVID is the most common. Relative to other countries, the United States has a higher proportion of patients with CVID, approximately 40.2%, compared to the Middle East (2.6%) and Africa (1.3%) [12]. These differences may also be related to differences in diagnostic availability, reporting methods, and general education on the immunodeficiency. However, even within the United States, CVID is very commonly underdiagnosed and underreported. Many cases of the immunodeficiency are found incidentally because the immunofixation and immunoglobulin measurement testing required is cumbersome, and some patients may be asymptomatic. Thus, the prevalence of the condition may be even higher than expected [13].

Here, we report the case of a 50-year-old male who presented to the emergency department with B symptoms such as weight loss, night sweats, and fever. After a biopsy of his disseminated lymphadenopathy, he was diagnosed with nodular lymphocyte-predominant Hodgkin lymphoma. Immunoglobulin analysis later revealed that he had low levels of IgG and IgM, findings consistent with CVID.

## Case presentation

A 50-year-old male patient with a past medical history of diabetes and hypertension presented to the emergency room with night sweats, weight loss of 40 pounds, intermittent diarrhea, and abdominal discomfort for the past six months. He also reported persistent subjective fever and occasional nausea.

A review of vital signs revealed that he was tachycardic at 108 beats per minute and had an elevated systolic blood pressure of 140 mmHg, while his diastolic blood pressure was 75 mmHg. However, he was afebrile and breathing room air with a normal respiratory rate. On physical examination, the abdomen was soft and nondistended with mild tenderness noted in the right upper quadrant as well as hepatomegaly and splenomegaly. Left cervical lymphadenopathy was palpable, and there was an enlarged supraclavicular lymph node that was firm and nontender.

Table 1 includes the set of vital signs and laboratory values obtained from the patient upon admission to further illustrate his clinical picture.

**Table 1:** Patient's vital signs and laboratory values upon initial presentation

Test	Result	Reference values
White blood cell count	3.63 x 10^3^ U/liter	4.5 x 10^3^ – 11 x 10^3^ IU/liter
Heart rate	108 beats per minute	60 – 100 beats per minute
Temperature (temporal artery)	36.5 °C	36.4 – 37.6 °C
Hemoglobin	11.1 grams/deciliter	13.2 – 16.6 grams/deciliter
Hematocrit	34.8%	37 – 52%
Platelet count	247 x 10^3 ^platelets	1.5 x 10^5^ – 4.5 x 10^5 ^platelets
Serum sodium	127 millimoles/liter	135 – 145 millimoles/liter
Serum potassium	4.7 millimoles/liter	3.6 – 5.2 millimoles/liter
Serum calcium	8.6 milligrams/deciliter	8.6 – 10.2 milligrams/deciliter
Serum chloride	92 millimoles/liter	96 – 106 milliequivalents/liter
Anion gap	11 milliequivalents/liter	4 – 12 milliequivalents/liter
Serum glucose	187 milligrams/deciliter	70 – 90 milligrams/deciliter
Aspartate aminotransferase	129 IU/liter	8 – 48 IU/liter
Alanine aminotransferase	109 IU/liter	7 – 56 IU/liter
Alkaline phosphatase	1233 IU/liter	44 – 147 IU/liter
Total bilirubin	1.8 milligrams/deciliter	0.2 – 1.2 milligrams/deciliter
Creatinine	0.8 milligrams/deciliter	0.74 – 1.35 milligrams/deciliter
Blood urea nitrogen	14 milligrams/deciliter	6 – 24 milligrams/deciliter

**Figure 1:**
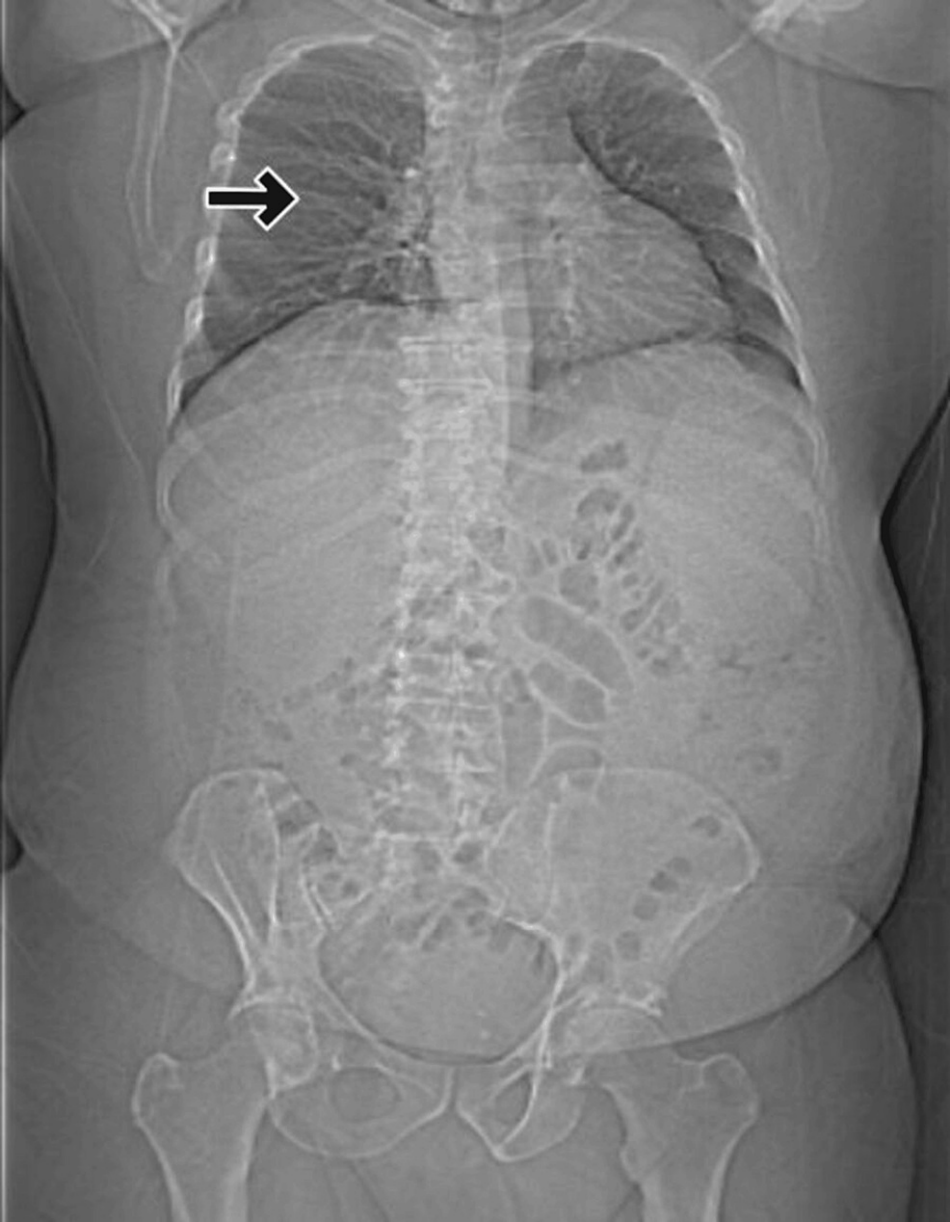
X-ray of the abdomen and pelvis with arrow demonstrating extensive lymphadenopathy

A chest X-ray (Figure 1) was negative for any acute chest abnormalities, and a limited abdominal ultrasound revealed hepatomegaly and hepatic steatosis. There was also an ill-defined hypodensity in the left hepatic lobe with adjacent prominent vessels. To obtain more information on the liver mass, a computed tomography (CT) scan of the abdomen and pelvis was performed. This revealed a hyperintensity in the left lobe of the liver with large enhancing vessels nearby, suggesting a possible vascular hemangioma. It also demonstrated splenomegaly with multiple diffuse infiltrated solid nodules consistent with lymphoma. There was also severe periportal, left supraclavicular, left internal jugular, peri-celiac, and retroperitoneal abdominal lymphadenopathy. Figure 1 demonstrates these findings. 

Given the presence of constitutional symptoms such as night sweats and weight loss in tandem with CT findings suspicious for malignancy, the patient was admitted to the hospital and a lymphoma work-up was performed. A lactate dehydrogenase level, human immunodeficiency virus (HIV) serology, hepatitis serology, complete iron panel, and D-dimer were ordered. The hepatitis and HIV panels later returned as negative.

To confirm the suspected diagnosis of lymphoma, interventional radiology performed an ultrasound-guided core needle biopsy of the left-sided supraclavicular neck lymph nodes, revealing pathology suspicious for lymphoma and secondary amyloid deposition. Amyloid stain (Congo red) was positive under green light. Bone marrow biopsy revealed that the patient had nodular lymphocyte predominant Hodgkin lymphoma confirmed by immunohistology positivity for CD20, CD3, and CD138 in small lymphocytes.

After diagnosis, immunoglobulin level measurements revealed that his IgG was low, IgM was low, and IgA was within normal limits. There was also negative serum immunofixation, a finding that did not support amyloidosis. The immunoglobulin levels can be found in Table 2 below.

**Table 2:** Patient's immunoglobulin level measurements

Immunoglobulin Type	Immunoglobulin Level	Reference Values
IgA	160 milligrams/deciliter	47 – 310 milligrams/deciliter
IgG	573 milligrams/deciliter	600 – 1640 milligrams/deciliter
IgM	43 milligrams/deciliter	50 – 300 milligrams/deciliter

The labs that showed low IgG and IgM with an elevated alkaline phosphatase were highly suggestive of common variable immunodeficiency. The patient was subsequently discharged from the hospital and was instructed to follow up in the outpatient hematology/oncology clinic while pending a full excisional lymph node biopsy.

## Discussion

This 50-year-old male presented to the emergency room with classic ‘B’ symptoms, including fever, night sweats, and a 40-pound weight loss. He was subsequently diagnosed with stage IV nodular lymphocyte-predominant Hodgkin lymphoma, a malignancy characterized by the abnormal proliferation of B and T cells.

Given the defects that many CIVD patients like this one have in key immunomodulators of B and helper T cells, they also have a higher likelihood of developing Hodgkin lymphoma. Most cases of Hodgkin lymphoma are diagnosed between ages 15 and 30 and can be further divided into the classical and nodular lymphocyte-predominant subtypes. Classical Hodgkin lymphoma always has Reed-Sternberg cells and is positive for CD15 and CD30. The nodular lymphocyte predominant variation is negative for both and usually positive for CD20 and CD45. This variation is more common than classical Hodgkin lymphoma and carries a good prognosis.

When suspected, lymphoma workup ought to include a complete blood count with differential, an erythrocyte sedimentation rate, and serum chemistries, including liver and renal function tests and albumin. HIV testing and hepatitis testing also ought to be performed. In our patient, all these laboratory tests were conducted with negative HIV and hepatitis results. Laboratory values were consistent with lymphoma, as our patient had leukopenia and anemia with elevated aminotransferases and alkaline phosphatase.

This patient’s core needle biopsy demonstrated eosinophilic extracellular slightly fibrillary material that was compatible with amyloid with the pathognomonic positive Congo red stain positivity. Amyloidosis is a systemic disease that occurs when the monoclonal light chains kappa (κ) or lambda (λ), which are normally soluble in the blood, form insoluble fibrils that deposit in various tissues [14]. Whenever Congo red stain positivity is found, the amyloidosis must be further characterized into its subgroup, as this guides treatment [15]. The test of choice is immunofixation, which is performed with immunohistochemistry. However, in our patient, immunofixation was negative for amyloid, leading us to believe that the initial finding of amyloidosis was incidental.

Given amyloid light-chain (AL) amyloidosis’ high rate of comorbidity with multiple myeloma and monoclonal gammopathy of undetermined significance (MGUS), this patient’s immunoglobulin levels were measured as well. He was found to have low levels of IgG and IgM with IgA levels that were on the lower end of normal. Thus, he met the criteria for diagnosis of common variable immunodeficiency [16].

Like in this patient, the diagnosis of common variable immunodeficiency can be a difficult one to make. This patient did not have the classic history of sinopulmonary infections, autoimmune disease, or bronchiectasis that many common variable immunodeficiency patients have. Thus, it was a diagnosis of exclusion, after amyloidosis and other medical conditions were ruled out.

The definitive treatment for common variable immunodeficiency is immunoglobulin replacement therapy, which reduces the burden of further infections by strengthening the patient's immune response [17]. 400 to 600 milligrams/kilogram of intravenous immunoglobulin are given every three to four weeks. The patient’s response to this treatment is measured, and the dose is increased if the patient continues to have recurrent bouts of respiratory infections or other complications.

The consequences of not diagnosing and treating common variable immunodeficiency early can be substantive. Had common variable immunodeficiency been diagnosed earlier in life, this patient could have been screened earlier for commonly associated pathologies, including lymphoma, gastric cancer, bronchiectasis, and autoimmune disease. 

## Conclusions

This case report described the clinical course of a 50-year-old male who presented with a history of night sweats, 40-pound weight loss, subjective fever, and nausea. Nodular lymphocyte-predominant Hodgkin lymphoma with extensive mediastinal, hilar, periesophageal, cervical, and left supraclavicular lymphadenopathy was diagnosed through a combination of CT imaging, immunohistochemistry, and bone marrow biopsy findings. Thus, serum immunoglobulin levels were measured, and this patient’s clinical findings of low levels of IgG and IgM indicated the presence of common variable immunodeficiency. Through this case report, the authors aim to draw attention to the importance of having high clinical suspicion for CVID whenever a patient presents with recurrent sinopulmonary infections and/or lymphoid malignancy. Detecting and diagnosing this immunodeficiency earlier in life can lead to decreased mortality and morbidity.

## References

[1] Yazdani R, Habibi S, Sharifi L, Azizi G, Abolhassani H, Olbrich P, Aghamohammadi A (2020). Common variable immunodeficiency: epidemiology, pathogenesis, clinical manifestations, diagnosis, classification, and management. J Investig Allergol Clin Immunol.

[2] Martinez-Gallo M, Radigan L, Almejún MB, Martínez-Pomar N, Matamoros N, Cunningham-Rundles C (2012). TACI mutations and impaired B-cell function in subjects with CVID and healthy heterozygotes. J Allergy Clin Immunol.

[3] Azizi G, Rezaei N, Kiaee F, Tavakolinia N, Yazdani R, Mirshafiey A, Aghamohammadi A (2016). T-Cell Abnormalities in Common Variable Immunodeficiency. J Investig Allergol Clin Immunol.

[4] Giovannetti A, Pierdominici M, Mazzetta F (2007). Unravelling the complexity of T cell abnormalities in common variable immunodeficiency. J Immunol.

[5] Barbosa RR, Silva SP, Silva SL (2011). Primary B-cell deficiencies reveal a link between human IL-17-producing CD4 T-cell homeostasis and B-cell differentiation. PLoS One.

[6] Oksenhendler E, Gérard L, Fieschi C (2008). Infections in 252 patients with common variable immunodeficiency. Clin Infect Dis.

[7] Verma N, Grimbacher B, Hurst JR (2015). Lung disease in primary antibody deficiency. Lancet Respir Med.

[8] Sperlich JM, Grimbacher B, Workman S (2018). Respiratory infections and antibiotic usage in common variable immunodeficiency. J Allergy Clin Immunol Pract.

[9] Mormile I, Punziano A, Riolo CA (2021). Common variable immunodeficiency and autoimmune diseases: a retrospective study of 95 adult patients in a single tertiary care center. Front Immunol.

[10] Salavoura K, Kolialexi A, Tsangaris G, Mavrou A (2008). Development of cancer in patients with primary immunodeficiencies. Anticancer Res.

[11] Resnick ES, Moshier EL, Godbold JH, Cunningham-Rundles C (2012). Morbidity and mortality in common variable immune deficiency over 4 decades. Blood.

[12] Modell V, Orange JS, Quinn J, Modell F (2018). Global report on primary immunodeficiencies: 2018 update from the Jeffrey Modell Centers Network on disease classification, regional trends, treatment modalities, and physician reported outcomes. Immunol Res.

[13] Popa C, Fischer R, Kurbitaeva S, Ravakhah K (2022). Common variable immune deficiency: an outpatient experience. South Med J.

[14] Ryšavá R (2019). AL amyloidosis: advances in diagnostics and treatment. Nephrol Dial Transplant.

[15] Melmed GM (2009). Light chain amyloidosis: a case presentation and review. Proc (Bayl Univ Med Cent).

[16] Ghafoor A, Joseph SM (2020). Making a diagnosis of common variable immunodeficiency: a review. Cureus.

[17] Kasztalska K, Ciebiada M, Cebula-Obrzut B, Górski P (2011). Intravenous immunoglobulin replacement therapy in the treatment of patients with common variable immunodeficiency disease: an open-label prospective study. Clin Drug Investig.

